# A sensation of COVID-19: How organizational culture is coordinated by human resource management to achieve organizational innovative performance in healthcare institutions

**DOI:** 10.3389/fpsyg.2022.943250

**Published:** 2022-09-29

**Authors:** Yingmin Zhang, Philip Saagyum Dare, Atif Saleem, Caleb Chidozie Chinedu

**Affiliations:** ^1^Zhejiang Provincial Education Examinations Authority, Hangzhou, China; ^2^Faculty of Education, Monash University, Melbourne, VIC, Australia; ^3^College of Teacher Education, Zhejiang Normal University, Jinhua, China; ^4^Faculty of Technical and Vocational Education, Universiti Tun Hussein Onn Malaysia, Johor, Malaysia

**Keywords:** human resource management, organizational culture, innovative performance, working environment, medical universities, healthcare institutions, pandemics

## Introduction

Globally, COVID-19 is a historic health epidemic that shook the entire world, causing immense dread and anxiety since its outbreak. The epidemic has had significant effects on economies, societies, workers, and institutions, including healthcare institutions. This situation began in December 2019 in Wuhan, China where the severe acute respiratory syndrome coronavirus 2 (SARS-CoV-2) first emerged. Its rapid spread and degree of health impact prompted the WHO to declare a global pandemic on 11 March 2020 (Hamouche, 2021).

Considering the rapid mode of transmission of the COVID-19 pathogen, several nations implemented a series of non-pharmaceutical countermeasures, including social isolation, to combat its spread. Quarantining people is one of the consequences of these measures, as are temporary closures of schools and universities, including healthcare education institutions and extraneous organizations, as well as travel restrictions, flight cancellations, and restrictions on large public and social events (Gourinchas, 2020; Brodeur et al., 2021; Hamouche, 2021). Such consequences in turn affected the smooth running of healthcare institutions and their grant functionality in terms of human resource management (HRM) staffing, training, performance, health, and safety management including handling relations of employees; organizational culture, and innovative performance.

Human resource management comprises the employment, management, and development of people within organizations (Armstrong and Taylor, 2020) and institutions where healthcare institutions are no exception. COVID-19 has had a considerable impact on healthcare institutions and organizations, posing critical challenges for HRM administrators and professionals, conventional organizational culture, and performance outcomes. Hamouche (2021) claimed that HRM, organizational culture, (OC), and innovative performance (IP) are all distinct concepts that are interdependent and that any changes to one will have an impact on the others. Therefore, the current opinion article presents a commentary (a talk/perspective) on ways that organizational culture is coordinated through human resource management practices to achieve organizational innovative performance to reinforce organizational transformation among healthcare institutions during the pandemic.

## Human resource management cogwheel

Human resource management has a strategic function that ensures organizational efficiency in human resources. The resources-based paradigm suggests that through HRM, the resources of organizations are managed to reinforce development and competitive opportunities for advanced performance, leading to an eventual increased competitive advantage in the viewpoint of Salas-Vallina et al. (2020). This theoretical paradigm establishes that organizational intrinsic strategic resources include abilities, procedures, information, and intellectual stimulation that enhance the development and maintenance of competitive opportunities.

Globally, the international market necessitates businesses to have a strategic view of intellectual stimulation (Carreiro and Oliveira, 2019). HRM is essential for encouraging innovation within organizations (Li et al., 2006) by impacting creative practices (Jiang et al., 2012) and intellectual systems (Jiménez-Jiménez and Sanz-Valle, 2011). Recent organizational studies confirm that firms pursue innovation because it ensures their businesses’ sustainability in an evolving environment (Acosta-Prado et al., 2020), i.e., the COVID-19 pandemic environment; the consequence of this vision is termed innovative performance. In order for online higher education institutions’ teaching models to be proactive in serving the demands of institutions, parents, and students, they must be redefined through innovation.

Organizational IP makes services more accessible and democratic (George et al., 2015) to its institutions, and employees and should be considered imperative in every institutional settings especially during the COVID-19 pandemic. Therefore, innovative performance gives an opportunity to diversify access to fundamental teaching services (Christensen et al., 2015) by implementing innovative solutions to maximize organizational change. Through performance innovation, healthcare institutions attain social innovation that results in better goals, which match teachers’, parents’, and students’ expectations (Phillips et al., 2015). A healthcare institution’s IP comprises new projects, methods, or services that address the different demands of students and introduction of novel concepts or procedures that result in enhancement of studies and instructions; thus, social innovation entails cooperation to design and carry out solutions to societal crises (Jaskyte, 2018, 2020; Brimhall, 2019) such as the pandemic crisis.

## Organizational culture

Organizational culture is shaped by the application of HRM’s tactics. Specifically, Padilha and Gomes (2016) claimed that an innovative culture may result in an innovative performance. Other studies further indicate HRM is the major pathway to achieving successful organizational performance within organizations, implying HRM can impact healthcare institutions’ OC (Aktar and Pangil, 2017; Baluch, 2017) during the pandemic’s organizational transformation. In addition, OC is a distinguished component that reinforces the dynamism of IP (Jaskyte, 2015; Meyer and Leitner, 2018). Various studies conducted on different contexts have also confirmed these relationships with strong findings (Brimhall, 2019; Narapareddy and Berte, 2019). Therefore, OC is expected to influence the IP of healthcare institutions both directly and indirectly through HRM. Healthcare institutions, besides the pandemic, encounter other counter-following challenges. Therefore, it is significant that institutions become innovative to enable them to achieve a successful transformation through HRM and OC.

## Innovative performance

Generally, the term innovation refers to new or considerably enhanced products, goods and services, procedures, modern marketing tactics (e.g., social media), or organizational style in corporate operations, workplace organizations, and external interactions. In view of this perspective, Aksoy et al. (2019) posit innovation as the capacity to produce and innovate as possibilities to address social needs, establishing structural restrictions and restoring innovation’s significance.

In the context of healthcare, Crespo-Gonzalez et al. (2020) consider innovation as the initiation of a new approach, concept, operation, or method in an attempt to reinforce treatment, assessment, education, protection, and research with a protracted goal of enhancing quality, security, outcomes, efficiency, and expenditures. This indicates that innovative healthcare methods have the potential to reduce death and morbidity rates. From a patient’s point of view, innovation in the healthcare sector means better care and less pain caused by an illness. This means there is a lot of room for innovation in these types of services.

As healthcare institutions are managed within the framework of health regulations, innovative performance from such institutions can be applied to healthcare services. According to Svensson et al. (2020), these kinds of innovations happen when institutions find better ways to solve a specific social crisis like the COVID-19 pandemic and encourage positive social change. IP can be affected by different factors, but Tataw (2012) suggests that HRM and OC are significant in certain sectors like healthcare.

## Human resource management and organizational culture

**FIGURE 1:**
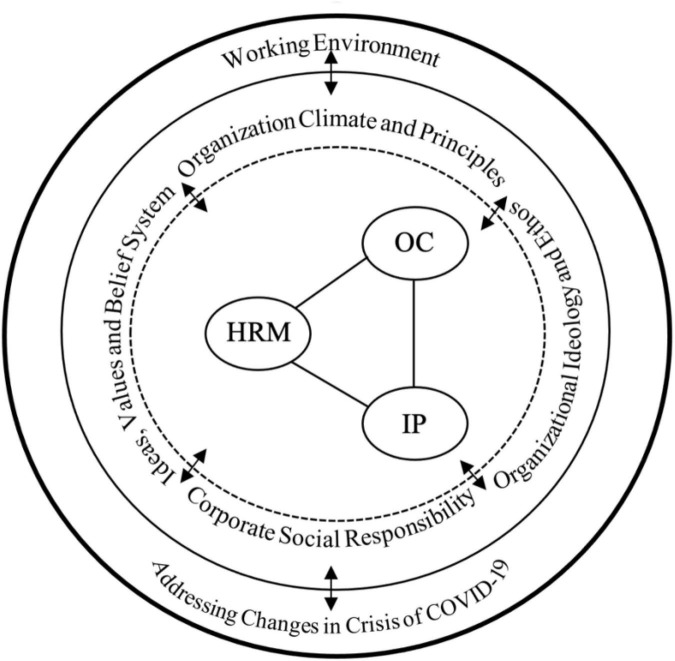
Working environment and epidemic crisis.

Organizational culture comprises the ideals, values, and behaviors of staff in an organization. Individuals’ perceptions of what is possible, as well as their morality and ethical rules, are directly related to their values (Pathiranage, 2019; Roscoe et al., 2019). Individuals’ opinions, which can be evaluated on a scale from true to incorrect, are referred to as their ideology. Significantly, individuals’ ideas and values play a direct role in the formation of their behaviors, which can be described as patterns of activities they engage in Wong et al. (2021). These behaviors and ideologies are crucial to their participation in organizational activities, processes, and strategies. Therefore, an organization’s ideas, values, and behaviors can become incorporated into an ethos or organizational ideology, which can then give guidance for managing the unpredictability of a tough or uncontrollable crisis such as the pandemic (Figure 1).

In the case of healthcare institutions, the demands of the pandemic imply that the leadership of institutions should incorporate the ideologies of their workforce toward pandemic management strategies to enable them to achieve a smooth organizational transformation during the pandemic. It is undeniable that the pandemic was met with several ideologies and people within various organizations including the healthcare sector may get engaged in activities within the framework of such ideologies. Organizational change process may have implication a successful transition through pandemic to manage the crisis (Figure 1), hence the need to establish an effective organizational culture reinforced by HRM.

An organization’s culture is formed when its guiding principles are acted out in the actions of its workers; eventually, those actions evolve into routines that are ingrained in daily operations of the organization (Roscoe et al., 2019). Healthcare institutions that ingrained the beliefs and ideas of their workforce in planning for pandemic management strategies may have a smooth transition compared to others. However, these cannot be achieved without appropriate HRM practices.

Human resource management is crucial in fostering an organizational culture since it influences ideas, values, and behaviors in the workplace through recruitment, development, evaluation, and incentive procedures (Amini et al., 2018). That is, in order for healthcare institutions to maximize a successful organizational change during the pandemic, they may have to recruit temporary staff to discharge certain roles such as temperature testing, cleaning, and sanitation staff among others.

As a matter of fact, a study that was conducted not too long ago by Pellegrini et al. (2018) highlighted the significance of constructing human resource practices in such a way as to improve employment, work engagement, and behavior to support organizational change that leads to everlasting sustainability (Pellegrini et al., 2018). As a holistic approach, sustainable management enables companies to become innovative by thinking outside the box and, hence, achieve versatile sustainable standards in the competitive industrial market (Aragón-Correa et al., 2022; Whittingham et al., 2022). In a prior study, Attaianese (2012) observed that professionals who were trained and given incentives to participate in organizational transformational practices ultimately helped the company build and nurture a culture all across the entire organization to attain resource sustainability.

## Role of encouraging organizational culture

Srinivasan and Kurey (2014) highlighted that a major change in the company culture of sixty United States-based multinational corporations was brought about by four criteria: emphasis on leadership, credibility of information, empowering the workforce; and engagement of peers. Applicably, healthcare institutions besides the ideas, values, and beliefs of their employees, may resort to a culture that emphasizes leadership, information credibility, workforce empowerment, and peer engagement in organizational activities in order to drive organizational change during the pandemic.

In spite of the fact that these characteristics are responsible for movement toward a quality management culture (Srinivasan and Kurey, 2014), we contend that they are also capable of enabling an organizational culture that promotes acute organizational transformation. Organizational culture functions as a bonding agent between staff and an organization’s system while also fostering positive and innovative behaviors in the workplace (Khan et al., 2018, 2020).

By fostering an environment where individuals’ thoughts and ideas are challenged, a culture of innovation fosters collectivism within groups. Mekpor and Dartey-Baah (2017) and Khan et al. (2018) assert that the HRM practices of institutional leaders aim to foster a culture of innovation by encouraging intellectual stimulation. A culture of innovation cannot exist without the backing of a resourceful HRM leader. Organizational innovation is characterized by an innovative, results-driven, creative, and demanding work setting that promotes HRM leadership (Yu, 2017). Despite the fact that previous research has demonstrated that varying factors primarily determine innovation, organizational culture and resourcefulness skills are influential factors that promote innovation within institutions (Rabbani et al., 2014), including healthcare and higher education institutions.

## Transition during the pandemic: Human resource management, organizational culture, and innovative performance

In the current COVID-19, a health care and medical instructions’ most significant characteristic is a dynamic HRM flexibility, structures, learning as well as sustainable innovative performance. To gain greater success, the healthcare education system today needs to be tailored to continual changes. Organizational HRM is the key method for increased adaptation (Jiang et al., 2012). The entire healthcare education system must be built on a very high standard of HRM and OC for effective output and results. Instead, in the modern century, leaders and administrators in healthcare instructions are being pushed to use more information to resolve confusion and sustain ongoing circumstances across evolving conditions. This requires that education administrators and leaders consider a high priority for the sustainability of organizational innovation and innovation management in healthcare institutions.

Implacably, the COVID-19 pandemic was a test for most institutional managers in healthcare and medical institutions, because they are required to tailor their services and processes toward maximizing transformational organizational change to address the needs during the pandemic. The unpredictable pandemic situation required institution managers and leaders to respond swiftly to change to empower their workforce by developing an efficient organizational culture that fosters change. For instance, it is indicated by Mekpor and Dartey-Baah (2017) and Khan et al. (2018) that the HRM practices of institutional leaders aim to foster a culture of innovation by encouraging intellectual stimulation and cannot be achieved without the backing of a resourceful HRM leader. Therefore, the bond between HRM and OC is significant to enable healthcare institutions to achieve innovation and innovative performance in response to change.

In addition, Pavlova and Saenko (2017) and Armstrong and Taylor (2020) suggest that HRM is a continuous improvement philosophy that provides scientific tools and skill sets for fulfilling institutions’ future and current expectations and requirements. As for preparing human resources for any service and productive organizations, the determining as well as most significant factors are educational entities and organizations. It has been stated in organizational literature that HRM is a dominant tool for sustaining organizations’ innovative performance and increases the competitive advantage (Browning et al., 2009; App et al., 2012; Collins, 2020; Harvey and Turnbull, 2020).

It has been pointed out by Ballesteros-Rodríguez et al. (2012) that culture defines how things are done as well as affects leaders in establishing objective and HRM practices. The process of establishing objective and HRM practices in institutions suggest that healthcare institutions require continual efforts to remind their employees; teaching and non-teaching staff including students about the institutional functioning in terms of beliefs, values, and ideology to prepare them for the pandemic working environment.

This involves preparing an institution and working resources for organizational change by tailoring their beliefs and values toward developing an organizational culture that is responsive to change. That is, HRM practices are essential to organizational transformation especially in situations such as the pandemic. As stated by Kang et al. (2007), in the value creation process, a significant part played by HRM exhibits novel practices for improved IP. Organizational culture is a significant element to sustain an innovative performance because it enables the learning environment in institutions and organizations.

A learning and supportive organizational culture activates the innovation of an organization in the present complicated environment. Innovation success is related with organizational innovation capabilities and OC (Leal-Rodríguez et al., 2014). A body of literature has determined the influence of HRM on OC (Hartog and Verburg, 2004; Aktar and Pangil, 2017). It means implementing strategies to build an environment where staff can impact innovative findings through appropriate knowledge storage, distribution, and acquisition. It has been pointed out by Ballesteros-Rodríguez et al. (2012) that culture defines how things are done as well as affects leaders in establishing objective and HRM practices.

The institutional environment in this situation can influence change so healthcare institutions need to pay key attention to the organization of the institutional environment to foster the process of change. Tailoring healthcare institutions’ environment to function on the beliefs and values of employees during the pandemic could foster OC that promotes IP instigated by HRM. It can therefore be said that HRM is very crucial in fostering organizational change, so empirical studies should be exclusively conducted to assess how HRM influences the OC of healthcare institutions.

## Conclusion

Indeed, HRM influences individual achievements in terms of skills, commitment, and other individual characteristics associated with innovation (Diaz-Fernandez et al., 2017). In other words, performance innovation, regarded as the potential of a corporation to acquire new services and outputs, is highly associated with HRM (Adnan et al., 2016; Diaz-Fernandez et al., 2017). It is evident that HRM actions, such as fostering an organizational culture that promotes innovation and allowing employees to continue their professional development, have an impact on staff and, subsequently, on IP (Liao and Huang, 2016; Gile et al., 2018; Meyer and Leitner, 2018; Acosta-Prado et al., 2020).

In reaching the expected IP, a significant part is played by OC (Jaskyte, 2018). Besides, organization climate and healthcare management’s role are recognized in the literature as OC elements that enable a sustainable IP (Meyer and Leitner, 2018). Sustainable innovative performance is crucial to the success of healthcare institutions, and organizations are required to incorporate social and ecological concerns in their corporative agendas for innovation toward sustainability. In this regard, it is recommended for future investigations to focus exclusively on how healthcare institutional leadership influences the OC of healthcare institutions to foster IP in times of a crisis. This would be necessary to contribute to the organizational literature related to the healthcare sector and healthcare institutions. In the process of innovation development, HRM plays an important part by influencing creativity and knowledge management system (Li et al., 2006; Jiménez-Jiménez and Sanz-Valle, 2011; Jiang et al., 2012). Along these lines, HRM outreaches a knowledge-based perspective concerning organizational capacities that are related to organizational culture and impact innovation success (Leal-Rodríguez et al., 2014). Generally, innovation requires organizations to invest money, which is not easy to count sometimes for healthcare institutions. In general, healthcare institutions should be pushed by the resources-based theory (Acosta-Prado et al., 2020) for taking all their resources’ benefits for innovative performance.

As an opinion article, this commentary review is limited to the authors’ viewpoints of the literature accessed. In this direction, an empirical study is recommended to be conducted to test how organizational culture is coordinated by human resource management to achieve organizational innovative performance in healthcare institutions.

## Author contributions

All authors listed have made a substantial, direct, and intellectual contribution to the work, and approved it for publication.
